# Current applications of antibody microarrays

**DOI:** 10.1186/s12014-018-9184-2

**Published:** 2018-02-28

**Authors:** Ziqing Chen, Tea Dodig-Crnković, Jochen M. Schwenk, Sheng-ce Tao

**Affiliations:** 1 Key Laboratory of Systems Biomedicine, (Ministry of Education), Shanghai Center for Systems Biomedicine, Shanghai Jiao Tong University, 800 Dongchuan Road, Shanghai, 200240 China; 2 Affinity Proteomics, SciLifeLab, KTH - Royal Institute of Technology, 171 65 Solna, Sweden; 3 School of Biomedical Engineering, Shanghai Jiao Tong University, Shanghai, 200240 China; 4 State Key Laboratory of Oncogenes and Related Genes, Shanghai Jiao Tong University, Shanghai, 200240 China

**Keywords:** Antibody microarray, Signalling, Drug mechanism, Clinical application, Systems biology, Technology advances

## Abstract

The concept of antibody microarrays is one of the most versatile approaches within multiplexed immunoassay technologies. These types of arrays have increasingly become an attractive tool for the exploratory detection and study of protein abundance, function, pathways, and potential drug targets. Due to the properties of the antibody microarrays and their potential use in basic research and clinical analytics, various types of antibody microarrays have already been developed. In spite of the growing number of studies utilizing this technique, few reviews about antibody microarray technology have been presented to reflect the quality and future uses of the generated data. In this review, we provide a summary of the recent applications of antibody microarray techniques in basic biology and clinical studies, providing insights into the current trends and future of protein analysis.

**Electronic supplementary material:**

The online version of this article (10.1186/s12014-018-9184-2) contains supplementary material, which is available to authorized users.

## Background

Antibody microarrays are built on immobilizing antibodies for a parallel analysis of multiple targets in a given sample [1]. Today’s antibody and affinity reagent-engineering methods have helped to advance the methodology [2, 3]. Antibodies and a variety of antibody derivatives have been used to build arrays, including nanobodies, single-chain variable fragments (scFvs) and fragment antigen-binding (Fab)-fragments [4]. In addition, phage display [5] and ribosome display [6], combined with advanced materials and bioinformatics development have being driving forces in recent years [7].

**Fig. 1:**
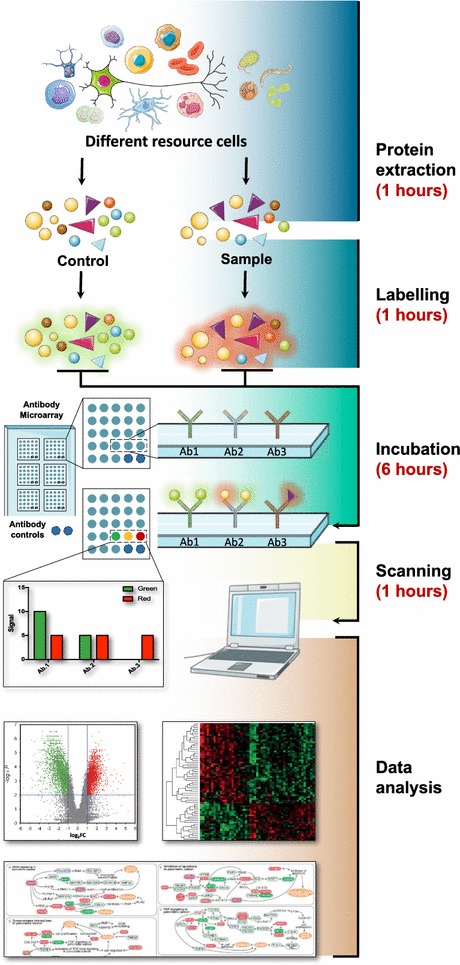
Review of planar antibody microarray technologies and their applications in the field of proteomics. Images were adopted from Servier Medical Art by Servier (http://www.servier.com/Powerpoint-image-bank) and modified by the authors under the following terms: CREATIVE COMMONS Attribution 3.0 Unported (CC BY 3.0)

The typical workflow of an antibody microarray is depicted in Fig. 1. Briefly, antibodies are immobilized onto a chemically functionalized or otherwise modified surface. After blocking the reactive groups of the surface, a sample containing soluble proteins of interest is incubated on the array, and the targeted proteins from the sample are captured by the antibodies. The resulting binding events are reported directly by fluorescent labelling of the sample or by the addition of a secondary detection reagent.

The attractiveness of antibody microarrays is that they can be used to study a diverse number of biological processes [8] and have been used to investigate protein–protein interactions [9], signal pathway analysis [10], studies of post-translation modifications [11], and detection of toxins [12]. In the clinical context, arrays have enabled opportunities to identify novel disease biomarkers [13] as well as generating unique proteome signature by comparing healthy and disease states. This information will be of great value in the future, enabling better disease management through improved diagnostics and the ability to track disease status and therapeutic efficacy.

**Fig. 2:**
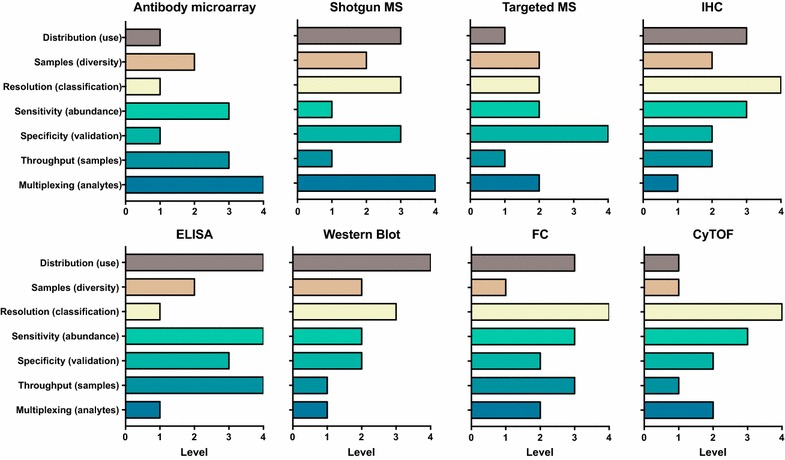
Comparison of different protein detection method following 7 categories: multiplexing (analytes), throughput (samples), specificity (validation), sensitivity (resolution), flexibility (content), samples (types), distribution (use). The scale number equal to high (4), medium (3), low (2), lower (1). The use of the scales as a comparative measure within each category. *IHC* immunohistochemistry, *ELISA* enzyme-linked immunosorbent assay, *FC* flow cytometry, *CyTOF* mass cytometry

Antibody microarrays have demonstrated a number of advantages compared to traditional, single analyte methods of protein analysis, such as, enzyme-linked immunosorbent assays (ELISA) and Western blotting. Microarrays are high throughput, highly sensitivity, require small sample volumes, and more recently have become more standardized and user friendly experimental procedures. Compared with mainstream proteomics strategies, especially mass spectrometry (MS), the process of antibody microarray assays is fast and takes less than 24 h from sample preparation to data interpretation. Detailed comparison is shown Fig. 2.

In theory, like DNA microarrays, antibody microarrays can be designed to host a few to thousands, or even ten-thousands, of features. Currently, high features have been achieved by immobilizing proteins [14] or lysates [15, 16], and antibody microarrays are under active technological development and to-date operate at a few hundred features [17–19]. The arrays can be constructed either host many features per sample or be designed to compartmentalize the array into sets of arrays that allow many samples to be investigated simultaneously. Generally, the latter is more common, particularly if a large number of clinical samples are analysed in a given study. For the analysis of a large amount of samples, bead based systems have become attractive, as they can be processed in microtiter plates and by liquid handling devices using cytometry for instant data availability [20, 21]. Importantly, antibody microarrays generate high dimensional data that can be processed using already well-established DNA microarray software, since the images scanned from planar antibody microarrays are similar to those of DNA microarrays.

Antibody microarray experiments are most often used as the initial tool during the biomarker discovery process. These results continue down the validation pipeline using additional antibody-based assays, such as ELISA and Western blotting, or immune-capture MS to validate the findings clinical/pathophysiological relevance [22]. However, the cross-reactivity to off-target proteins that are more abundant than the target of interest is still a challenge for the single binder version of the assay. Thus, undesired reactions with other target proteins represent one of the main obstacles in establishing high performance and high specificity assays. The scope of this review is to outline the antibody microarray techniques that are being used today for biology studies and clinical research.

## Recent applications (since 2011)

Due to unique capabilities of the antibody microarray and its applicability in a range of biomedical projects, a series of different antibody microarrays have been developed, of which some have become commercially available. Herein, we have collected some of the representative studies in the last 7 years (Table 1), and organized our overview into two parts depending on the focus of the projects: basic biology-driven studies and clinical research. As it remains practically impossible to cover all the recent applications of antibody microarray, we focus on those applications which we deemed are of high interest and findings of important clinical significance.

**Table 1:** Antibody microarray summary

Lab/company	Area	Antibody microarray type	Abs number	PMID
Medsaic Pty	Colorectal cancer	DotScan™ CRC microarrays	122	25445327
Medsaic Pty	Systemic lupus erythematosus	DotScan™ antibody microarray	82	23516448
Medsaic Pty	Chronic lymphocytic leukemia	DotScan™ CLL antibody microarray	182	24289109
Medsaic Pty	Cancer	DotScan™ antibody microarrays	144	27086589
Clontech	Alzheimer’s disease	Antibody Microarray 500	500	22554416
Clontech	Prostate cancer	Antibody Microarray 507	507	23280553
Clontech	Glaucoma	Ab Microarray 500	508	22974818
Fullmoon Biosystems Inc		Explorer Antibody Microarray	656	
Fullmoon Biosystems Inc	Squamous cell lung and esophageal carcinoma	PEX100	1318	26040563
Fullmoon Biosystems Inc	Prostate cancer	Phosphorylation-specific antibody microarray	95	24009409
Fullmoon Biosystems Inc		Customized antibody microarray	248	
Kinexus Bioinformatics	Smoke	KAM-1.0	650	21627322
Kinexus Bioinformatics	Breast cancer	KAM-1.1	650	21423216
Kinexus Bioinformatics	*A. pleuropneumoniae* infection	KAM-850	854	26577697
Kinexus Bioinformatics	hiPSC-CMs	KAM-850	854	25055963
Sigma-Aldrich	Breast cancer	Cell Signaling Antibody Microarray	224	21394501
Lab Vision Corporation	Programmed cell death	TAA-001 Lab Vision Corporation	720	23619569
Abnova	Alzheimer’s disease	Master Antibody Microarray	656	25428253
Proteogen Inc	Drug mechanism	ProteoChip	48	21558493
Jörg D. Hoheisel	Non-muscle-invasive bladder cancer	Home made	818	24610664
Jörg D. Hoheisel	Glioblastoma	Home made	724	26232108
Jörg D. Hoheisel	Pancreatic cancer	Home made	810	22579748
Paul D. Lampe	Pancreas cancer	Home made	4096	25589628
Paul D. Lampe	Biomarker for cancer	Home made	3600	24185138
Paul D. Lampe	Autoantigen-autoantibody complexes	Home made	3600	23541305
Nicholas A. Shackel	Liver diseases	Home made	60	22863037
Nicholas A. Shackel	Hepatitis C virus	Home made	90	25706280
Christer Wingren	Pancreatitis	Home made	121	22930578
Diane M. Cibrik	Renal allograft rejection	Home made	108	24323459
Daniel Böhm	Breast cancer	Home made	23	21885915
Carl Borrebaeck	Pancreas cancer	Home made	180	25196118

Review of planar antibody microarrays from recent studies. Lab/company, area of research, antibody microarray type and number of immobilized antibodies (abs) are summarized. The microarray with the highest number of antibodies belongs to Paul D. Lampe’s group and their home-made antibody microarray

### Basic research

#### Signalling pathway related

Changes in signalling pathways is a hallmark of many disease states, including cancer [23, 24], diabetes [25, 26] and neurodegenerative disorders [27]. Building antibody arrays using capture reagents targeting proteins in the signalling cascade of interest has enabled researchers to investigate changes in protein profiles and modifications in signalling pathways in normal biological processes and disease states. Researchers have applied this concept to a range of biological sample materials and preparations, including cell lysates [28], tissue extracts [29], and plasma [30].

Calbindin-D28 k (CB), an important calcium-binding protein that acts as a calcium buffer and is found to be expressed at lower levels in the brains of mice and humans with Alzheimer’s disease but it is not known if these changes contribute to AD-related dysfunction (AD). Kook et al. generated a CB-deficient Alzheimer’s transgenic mouse (CBKOTg) to investigate in CB’s contribution to signaling pathways in AD [23]. Utilizing antibody microarrays to examine mouse brain tissue, they identified significant alterations in cell death pathways, synaptic transmission and MAPK signalling pathways upon CB deletion. These findings were verified by immunohistochemistry which showed increased apoptotic markers and increased neuronal death, demonstrating that antibody microarrays can provide novel information, enhancing our understanding of the role CB and its implication in AD pathophysiology.

Antibody microarrays have been used to uncover the physiological role in highly conserved proteins, including elongation factor 4. Gao et al. dissected the mechanism of quality-control factor mitochondrial Elongation factor 4 (mtEF4) in translation using a phospho-explorer antibody microarray [31]. By comparing cell lysates between mtEF4 knock-out and WT mice testis tissues, they found that the fold enrichment of mTOR was the highest among different signalling pathways. The authors showed that with mtEF4 deletion, the major feedback signal from the somatic cytoplasm is mTOR upregulation. This was accompanied by an increased cytoplasmic translation, thus indicating that mTOR plays a crucial role as a downstream effector compensating for mitochondrial translation deficiency [32]. Furthermore, the study linked previously undescribed cross-talk between mtEF4-dependent quality control in the mitochondria and the mTOR pathway in the cytoplasm.

#### Drug mechanism

With the help of antibody microarray assays, researchers can investigate drug mechanisms in a systematic and efficient manner. Among many possible applications, tumor-induced angiogenesis has been studied as it plays a pivotal role in cancer progression [33]. P11, a novel peptide ligand containing a PDZ-binding motif (Ser-Asp-Val) with high affinity to integrin α_v_β_3,_ was identified from a hexapeptide library (PS-SPCL) using an antibody microarray [34]. The pharmacological mechanism of P11, was elucidated using a specifically designed pharmacoproteomic microarray approach containing 48 cancer related antibodies [35]. The study revealed that P11 inhibits bFGF-induced human umbilical vein endothelial cell proliferation via mitogen-activated protein kinase and extracellular-signal regulated kinase inhibition. In addition, the microarray revealed P11 caused the upregulation of apoptotic marker p53, resulted in apoptosis induction via activation of the caspases system, which indicates P11 may play a key role in preventing tumor progression. The link between P11 and p53 presents clinical and basic cancer researchers will help to elucidate further clues about the role of P11 and its inhibitory target.

Lovastatin, a natural product derived from *Aspergillus terreus* or *Monascus ruber*, has been widely used as a cholesterol-lowing drug in the clinic. Yang et al. have used an antibody microarray containing 656 antibodies with a focus on different functional cell pathways, which suggested that lovastatin also has anti-cancer properties, through poorly defined mechanisms. There is a strong need to find effective cancer treatments and to understand their mechanisms of action. Using a microarray, breast cancer cells were studied under hypoxic conditions to investigate the molecular mechanisms through Lovastin exherts its effect. They showed 17 up-regulated proteins and 20 down-regulated proteins for lovastatin treated breast cancer cells, compared to the control, and the results were subsequently validated by real-time PCR [36]. The protein signature included proteins involved in apoptosis, cell proliferation and tumour metastasis, linking modulation of these pathways to the pharmacological action of lovastin.

The type 2 diabetes drug metformin, has also proposed to have anti-cancer properties. Lee et al. investigated the potency of novel metformin derivatives using an antibody microarray built to study cell cycle-related molecules. The authors showed that metformin-butyrate (MFB) had better anti-tumor efficacies than metformin-HCl, through greater neoplastic activity and better efficiency at impairing cell cycle progression during S and G2/M phases in addition to appearing to have preferential cytotoxic effects on breast cancer stem cell populations [37]. These findings highlight how antibody arrays can provide proteomic evidence supporting development of drugs with better efficacy.

For basic biology research, antibody microarrays can serve in detecting signalling proteins and proteins associated to a particular phenotype. Arrays with parallel protein detection create an opportunity to investigate multiple signalling pathways in a single experiment, providing insights into novel mechanisms, such as the study of disease progression, drug interaction, and response to infections.

### Clinical research

Analysis of clinical samples at the protein level will enable improvement in diagnostics markers, and directing treatment options. Since proteins are involved in the majority of cellular processes, protein analysis may provide a biological disease signature, generating detailed insights about the current state of a cell, organ or system. Antibody microarrays can provide a real-time guide of current changes in biological processes during health and disease. In this section, we will exemplify how antibody microarrays have been used to investigate clinical cohorts, primarily analysing serum and plasma samples. It is not possible to cover all the aspects of antibody microarrays for clinical research, herein, we only focus on several of the most important diseases, i.e. autoimmune diseases, infectious diseases, cancer and neurodegenerative diseases.

#### Autoimmune diseases

The diagnosis of systemic lupus erythematosus (SLE) is challenging due to its heterogeneous clinical presentation and the lack of robust biomarkers to distinguish it from active and inactive disease as well as from other autoimmune diseases. Lin et al. [38] performed a DotScan™ antibody microarray screening of peripheral blood mononuclear cells from 60 SLE patients of varying disease activity, 25 rheumatoid arthritis patients, 28 other autoimmune disease samples, and 24 healthy controls. The antibody microarray profiles could distinguish active SLE patients from healthy controls. Using a leukocyte capture array improved the discriminative ability of conventional SLE diagnostics, by verifying serum anti-dsDNA, complements C3 and C4, the microarrays increased the capability of discrimination of semi-active and active SLE, information which will assist better disease management.

In another study, Carlsson et al. [39] constructed 135 human recombinant single-chain fragment variables (scFv) targeting immune proteins to build an in-house antibody microarray. In this study, they examined patients with systemic sclerosis (SSc), SLE and 15 healthy volunteers. SSc is an autoimmune disorder affecting connecting tissue, which can be challenging to differentially diagnose from SLE. The array identified 40 differentially expressed proteins creating a candidate proteome signature to delineate SLE and its severity from SSc. This protein signature was a better disease classification than single or even combinations of conventional clinical parameters, including, ANA, anti-DNA, SLEDAI-2 k, C1q, C3, C4 and CRP, illustrating the potential use for antibody microarrays to create new disease-signatures which will add clinical value to disease management.

#### Infectious diseases

Infections activate the immune system differently, and these mechanisms can be investigated using antibody arrays on a proteome-wide level. *Helicobacter pylori* is a pathogen that colonizes roughly one half of the world’s population which causes chronic gastritis. Sukri et al. [40] employed the DotScan™ antibody microarray to determine the tolerance of immune system toward tumor cells in gastric cancer using 144 CD antibodies to profile the distribution of CD markers between *Helicobacter pylori* infected and un-infected gastric adenocarcinoma cells. Interestingly, they found gastric adenocarcinoma cell line AGS infected by cagA + *H. pylori* showed increased CD27 expression, which is essential for maintenance of T cell population, and increased CD markers were also detected in *H. pylori*-infected gastric cancer patients. This study suggests not only the tolerance of the immune system toward gastric cancer, but also the immune response variations exploited by different *H. pylori* strains.

Furthermore, antibody arrays can be useful in tracking a continuing change in the physiological environment, including disease status and response to therapies and interventions. After liver transplantation, 94% of patients were found with hepatitis C virus (HCV) induced histological damage, with some patients suffering severe disease recurrence [41]. There is an urgent need for predictive biomarkers to identify recurrent disease severity. Using a CD antibody microarray, peripheral blood from patients defined as pre-transplant, early, mid, and late-transplant groups were investigated to predict the severity of HCV recurrence after transplantation [42]. Serial blood samples taken from patients before and after liver transplant enabled tracking of the disease milieu, with its own internal controls. Five CD markers (CD27, CD182, CD260, CD41, and CD34) were significantly increased in severe recurrence compared to mild recurrence. This result shows that antibody arrays may assist in assessing recurrent HCV disease severity after liver transplantation.

Ellmark et al. [43] devised a single framework recombinant antibody (SinFabs) microarray containing 127 different antibodies against immune-regulatory antigens selected from the n-CoDeR library on gastric adenocarcinoma. They elucidated that distinct tumor- as well as infection-associated protein expression signatures, such as IL-9, IL-11, and MCP-4, could be identified from the plasma proteomes and serve as potential biomarkers. These findings may aid to improve the understanding of *H*-*pylori* induced cancer and may pave the way for refined diagnostics in the future.

Bead based antibody arrays have been performed to investigate differential protein profiles in plasma in children suffering from malaria and malaria-related complications [44]. From 1000 proteins screened, 41 proteins had differential expression between malaria infected children and community controls. Thirteen further proteins were linked to malaria-disease severity. These findings indicate the involvement of inflammation as well as unbalanced metabolism of glucose in severe forms of the disease and the biggest changes were seen in two of muscle proteins (carbonic anhydrase 3 and creatine kinase) pointed at muscle damage and lesions for children with cerebral malaria. These findings may aid the development of simple tests for classifying malaria-infected children into group showing a higher risk for the severe form of the disease.

#### Cancer

Antibody microarrays have been applied for cancer research mainly to study cancer progression and candidate proteins that may serve as diagnostic biomarkers [45, 46]. Pancreatic cancer is an aggressive disease with poor prognosis, and disease-specific biomarkers that offer early and accurate diagnosis are in urgent need. Using an in-house developed recombinant antibody microarray platform, Wingren et al. [47] screened sera from 148 patients with pancreatic cancer, chronic pancreatitis, autoimmune pancreatitis (AIP), and healthy controls. They identified a panel of 25 protein targets, including IL-2, IL-11, IL-12, TNF-, which contributed to distinguishing pancreatic cancer from healthy controls. This 25 protein signature exhibits a high diagnostic potential (AUC of 0.88). In addition, the group has recently extended their analysis of their panel to additional sample sets, including lymphoma [48], prostate cancer [49], and breast cancer in cell lysates [50] and plasma [51]. These findings are useful to aid the development of novel and multivariate diagnostics methods.

One of the most important questions in cancer biology, is determining the invasiveness of a tumor. One study developed a customized an antibody microarray platform containing 4096 features to interrogate plasma samples spanning pre-invasive and invasive diseases from a mouse model of pancreatic ductal adenocarcinoma (PDA) [18]. They found a protein signature, comprising of the differential expression of three proteins, ERBB2, TNC and ESR1, which could be used to improve the AUC from 0.86 (95% confidence interval [CI] 0.76–0.96) to 0.97 (95% CI 0.92–1.0) when the PDA marker CA19-9 included.

The majority of newly diagnosed cases of bladder cancer are low-stage, low-grade, non-muscle-invasive [52]. After standard transurethral resection, 50–70% of tumors recur, however 10–30% of the tumors will progress to muscle-invasive disease [53, 54]. Understanding the mechanism of tumor progression could provide more useful information to clinical scopes. Srinivasan et al. studied cell lysates with an antibody microarray containing 810 cancer related antibodies constructed by the Hoheisel lab [55]. The authors built a multivariate classifier containing 20 proteins, which facilitatated the prediction of recurrence with a sensitivity of 80% and a specificity of 100%. Interestingly, they found repression of the TGF-β signaling pathway in recurrent cancer. The signaling factors IFNG, TNF-α, and THBS1 were expressed less and the abundance of the inhibitor MAPK3 (also known as ERK1) was higher, while SMAD2, SMAD3, and SMAD4 were again significantly underrepresented [56]. The data indicated that TGF-β signaling pathway inhibitors may reduce bladder cancer recurrence [57].

Puig-Costa et al. [58] employed antibody microarrays to discover biomarkers for gastric cancer (GC). Notably, the antibody microarray contained antibodies targeting different well-known functional proteins that play a crucial role in cancer progression, including 120 cytokines, 43 angiogenic factors, 41 growth factors, 40 inflammatory factors and 10 metalloproteinases. Ingenuity pathway analysis confirmed that some biomarkers, such as ICAM-1 and angiogenin, indicated high inflammatory response in GC patients. Cellular movement and immune cell trafficking targets such as monocyte chemoattractant protein (MCP)-1 was found to be overrepresented within GC patients. The positive predictive and negative predictive values in this validation cohort were 75% (95% CI 53–90) and 80% (95% CI 56–94), respectively. Finally, antibody microarray analyses of the GC-associated inflammatory proteome identified a 21-protein inflammatory protein-driven gastric cancer signature (INPROGAS) that accurately discriminated GC from noncancerous gastric mucosa, and may provide new leads for the analysis of cancer progression.

In prostate cancer, Schwenk et al. [59] used antibody arrays on suspension bead arrays to compare plasma levels of proteins between different groups in order to find additional biomarkers alongside prostate-specific antigen (PSA). Besides classifying the patients based on PSA, they identified decreased plasma CNDP1 levels and in a subsequent larger studies this was shown to association in more aggressive forms of the disease. A sandwich immunoassay was developed to validate these findings in more than 1200 patients, and the association of the decreased CNDP1 to lymph node metastasis was further elucidated [60]. This demonstrates one of the very few studies that have successfully validated an initial indication through the antibody microarray pipeline and the development of a targeted assay. In a subsequent investigation of cancer cachexia, plasma levels of CNDP1 was found to be decreased in cachexic patients, hence pointing at a metabolic role of changes in CNDP1 levels [61].

In a study of small intestine neuroendocrine tumours, Darmanis et al. [62] investigated the plasma levels of two independent study set (77 and 132 samples) and a targeted bead array for 124 unique proteins. They could achieve classification accuracy of up to 85% using a panel of proteins and concluded with proposing novel candidates for classifying the tumors. Of the shortlisted candidates, such as for IGFBP2 and IGF1, the group performed ELISA assays to confirm the indications. These findings indicate a metabolic association of plasma proteins with cancer and support to consider these targets in future cancer studies.

#### Neurodegenerative diseases

Analysis of protein expression provides a possibility to extend the current knowledge on neurodegenerative pathophysiology [63]. Recent advances within the field of proteomics have offered the potential to search for novel biomarkers using antibody microarrays [64]. Advancement in protocol development have allowed researchers to study not only blood, but also cerebrospinal fluid (CSF) [69].

Within neuroproteomics, the Nilsson lab has conducted several studies by using antibodies form the Human Protein Atlas [65] together with the suspension bead array assays. Profiling CSF from patients with Multiple Sclerosis (MS), they found GAP43, a cytoplasmic protein involved in the formation, and regeneration of neurons, hence a promising biomarker diseases of the brain [66]. More recently, Remnestål et al. [67] compared protein levels in 441 CSF samples collected from different neurodegenerative disease sample sets as well as CSF collected post-mortem. Among 376 antibodies, the synaptic proteins GAP43 and NRGN were found to be associated to AD patients compared with controls.

Using plasma samples, CSF and brain tissue from patients suffering from MS, a large-scale screening was conducted that started from utilizing 4500 antibodies on bead arrays [68]. A set of proteins was found to be associated with MS subtypes in CSF and plasma. Utilizing some of the candidate antibodies raised against IRF8, IL7, and METTL14 also for immuno-fluorescence analysis of brain tissue showed staining of neurons in proximity of MS lesions. This indicates that antibodies selected from array-based assays for analysis of body fluids can also provide further evidence at the affected tissue.

Lastly, the bead arrays were used to profile plasma from patients suffering from amyotrophic lateral sclerosis (ALS) [69], where 367 ALS patients and 101 controls were analyzed for 278 proteins. The study concludes with proposing neurofilament medium polypeptide (NEFM), solute carrier family 25 (SLC25A20), and regulator of G-protein signalling 18 (RGS18) as valuable proteins because they are involved in processes related to disease pathophysiology, which warrant further validation in independent sample sets.

## Current issues and solutions

We list commercial antibody microarrays in Table 2, and further details about these products can be found as supplementary informtion (Additional file 1: Table S1). Despite the rapid technological advances in recent years, there are still technical issues that need to be overcome to ensure high-specificity and reproducibility of antibody arrays, to ensure high impact data and meaningful conclusions.

**Table 2:** Summary of commercial available antibody microarrays from different companies, Type G (Glass), M (Membrane)

Provider	Ab microarray category	Type	Max number of abs	Major focus
Raybio	215	G/M	400	Immune response, cytokines, cancer, signalling
Full moon	43	G	1358	Signalling, cancer biomarker, cytokines
Abnova	39	G/M	247	Signalling, cytokines
R&D	20	M	105	Signalling, cytokines, functional assay, proteomic profile
Abcam	14	M	400	Signalling, cytokines, functional assay, biomarker
MyBiosource	4	G	656	Signalling, proteomic profile
Hypromatrix	3	M	400	Signalling, functional assay
Panorama	2	G	112	Gene regulation, signalling
Arrayit	1	G	380	Plasma protein
Kinex™	1	G	878	Signalling

There is a need for validated antibodies for antibody microarray applications and respective sample types. The data from such validation efforts should consequently be provided. The quality of validated antibodies, the standardization of data analysis, and data storage and sharing are three important challenges to be considered.

Proteomic investigations using antibody microarrays have provided valuable data that reveal the pathophysiological background of a disease [70]. But there are also issues to address. Besides the overall still relatively small number of studies, the number of samples analysed in each study is usually limited to less than a hundred. Typically, these experiments are performed in an “exploratory cohort” first, examining preferably 1000’s of different features. These microarray results need to be validated with an independent set of preferably larger number of specimens and by other methods.

Moreover, samples prepared from frozen or fresh samples may provide different profiles depending on the specific set and that could be referred to a “study markers”. Another challenge of applying antibody microarrays for further studies is the heterozygosity of specific diseases (e.g. relapse remitting multiple sclerosis versus non-relapse multiple sclerosis) requesting careful study designs, in-depth knowledge about the sample itself and the disease phenotype of the subject it was obtained from. Some of these considerations concerning the design of discovery assays for the analysis of plasma proteome have been recently addressed elsewhere [71–73].

Finally, for a better study applying antibody microarray. Control should be carefully taken into consideration. Sample controls, not only internalize control like beta-actin or GAPDH for each sample, but also positive (or negative) control that already confirmed by other method would help to set cut-off for further analysis.

### The reproducibility challenge of the antibody microarray

One challenge for data derived from antibody microarrays is often linked to a lack of reproducibility. There are several experimental factors that may contribute to these problems, such as the surface chemistry and the mode of antibody immobilization, lack of sufficiently stringent processes for generation and validation of antibodies, as well as a bias from sample preparation, labelling, and batch effects from analysing samples across several slides or plates. We next focus on how to use general and novel methods to reduce systematic errors and potentially improve the reproducibility of antibody microarray experiment.

In the single-color approach, labels can be introduced at different efficacy in different samples due to chemistry dye binding stronger to certain amino acids. This might expand since the detection of proteins may reveal different fluorescent signal even though their quantity is the same. The samples could be labelled by a single fluorescent reporter dye, e.g. Cy3, Cy5, then probed one by one on antibody microarrays. In this circumstance, the slide-to-slide variation is unavoidable, thus reducing the reliability of the final microarray data and introducing systematic error. This systemic error can be attenuated by employing a dual-color labeling strategy inspired by DNA microarrays. Schroder et al. [74] reported an antibody microarray containing 741 cancer-related antibodies, employing dual-color mode. This approach has greatly improved the reproducibility, as reported in the study. The coefficients of variation (CV) of 89% features between 20 slides derived from the five production batches were under 15%, the CV of 96% features were under 20%. This dual-color mode can be applied in many other protein microarray experiments as long as they are comparing paired samples. This dual-color strategy will not only enhance the reproducibility of antibody microarrays, but may also improve the robustness of the assay.

### The cross-reactivity of antibody microarray

The DNA microarray has rapidly been scaled up from 256 to 6.5 million features, but to date sensitive antibody microarray assays have only been scaled up to thousands of targets [75]. Cross-reactivity, commonly arising when multiple detection antibodies are mixed, is a known weakness of these assays that is mitigated by lengthy optimization [76].

In antibody co-localization microarray (ACM) described by Pla-Roca et al. [77], both capture and detection antibodies were physically spotted onto the same two-dimensional coordinate, which reduced cross-reactivity and improved antibody reproducibility. Specifically, after spotting of the capture antibodies, the chip was removed from the arrayer, incubated with the sample, and then placed back onto the arrayer to be spotted with detection antibodies. After 3 years, the same group developed the next generation ACM, employing an array containing 50 capture antibodies. Instead of spotting, they used another perfect matched array with pre-spotted detection antibodies to transfer detection antibodies onto the assay slide. This method employs a pair of antibodies to detect a single protein similar to ELISA, thus improving the accuracy of the test that sets the path for an improved reproducibility.

### The availability of validated antibodies for antibody microarray

The primary power of antibody microarrays based on single binder assays lies in the capability of multiple protein detection. Theoretically, the power is only limited to the number of antibodies on the microarray. However, unlike DNA microarrays where the probes can easily be synthesized at large scale either off-chip or on-chip, antibodies have to be produced and validated one-by-one. Thus, the number of qualified antibodies for antibody microarrays is still limited and remains a challenge. To address this, high-throughput screening strategies such as phage display may hold the solution to this problem.

In addition, binding molecules of non-protein scaffolds have been described [78], yet the number remains low when compared to almost 3 million commercially available antibodies (http://www.antibodypedia.com/). However, the size of synthetic binder libraries can further be increased by introducing additional modifications to the scaffolds, such as aptamers [79]. To maximize the use and impact of affinity reagents, the EU FP7 programme AFFINOMICS (http://www.affinomics.org), together with the preceding EU programmes ProteomeBinders and AffinityProteome, aims to extend affinity proteomics research by generating a large-scale resource of validated protein-binding molecules for characterization of the human proteome [80].

Despite antibody use, a comprehensive scientific framework for the validation antibodies across research applications (i.e. immunohistochemistry, immuno-precipitation) is essential for the success of the antibody microarray platform. Uhlen et al. [81] have developed a five ‘pillars’ strategy for antibody validation: (1) genetic strategies, (2) orthogonal strategies, (3) independent antibody strategies, (4) expression of tagged proteins, and (5) immunocapture followed by MS for antibody validation. Immunoreagents, such as polyclonal, monoclonal antibodies, and other recombinant or synthetic binders are also suitable for this proposal. It will highly improve the quality and reproducibility for methods, such as antibody microarrays, given that the binders are validated in the intended application and for the sample type of interest.

For antibody microarrays, immuno-capture mass spectrometry or alternative assays such as dual-capture assays [82] offer similar assay formats for the target enrichment, as both require the immobilization of the binder. In the dual-capture system, antibodies are used to enrich their target proteins from a solution (e.g. plasma) and after labelling the proteins on the capture bead surface, the eluted proteins are detected by a multiplexed array consisting of several antibodies of interest. This allows two antibodies to bind a common target, hence presenting a sandwich assay based on consecutive binding. In comparison to MS, the antibodies used for read-out in affinity based assays need to be selected in relation to the target of the primary enrichment. Affinity assays are often still more sensitive and require less antibody and sample material then MS, however an MS based read-out will provide a wider view on which proteins have been enriched from a give sample, such as cell lysates [83] and more recently also plasma [84]. The breadth of MS data generated from such immuno-enrichment assessments requires though in order to carefully define specific peptides of interest from those that appear as unspecific and common contaminants [85].

### Data processing, analysis and storage

Unlike the DNA microarray community, which has established standards for data submission and storage, such as minimum information about a microarray experiment (MIAME) [86] these guidelines have not yet been implemented for antibody microarrays. Most of the analysis tools in use for proteome-analysis have been adopted from the field of DNA microarrays. The data formats are similar between antibody microarrays and traditional DNA microarrays, the data analysis can also be subdivided into steps of image capture, data preprocessing, differential expression detection, clustering. There are now several R packages available to support some of these steps, such as Limma [87], Clusterprofiler [88], Qlucore Omics Explorer (http://www.qlucore.com) and IPA (http://www.ingenuity.com). DNA microarray-specific MIAME standards have been applied in Gene Expression Omnibus (GEO), which includes some data sets from antibody microarrays. Since diversity of antibody microarrays exceeds those of DNA microarrays due to diversified applications, a classification scheme that can include different types of antibody microarray data is needed.

In data acquisition and analysis, Ensink et al. [89] provided an advanced approach for locating signals in microarray image data, called segment and fit thresholding (SFT). This approach was optimized based on the initial settings by locating background and signal on the acquired antibody microarray image and immunofluorescence data. It was found that SFT performed well over multiple, diverse image characteristics without readjustment of settings, promoting full automation in microarray image analysis.

Image acquisition parameters such as the laser power and photomultiplier tube gain (PMT) during scanning can influence the readout of fluorescent intensities and thus may affect data quantitation. Gu et al. demonstrated an experimental approach using two fluorescent dyes to determine optimal settings of scan parameters for microarray experiments. Their efforts may facilitate the improvement of the accuracy of quantitative outcome in antibody microarray experiments [90].

Several main technical features and assay procedures remain to be improved. The handling of protein microarray data, i.e. the biostatistics parts, is one of the key features. Delfani et al. [91] have standardized the analytical workflow of their in-house designed recombinant antibody microarray platform and Hong et al. [92] have described an approach of handling data generated from different assay batches from suspension bead array assays. This is an important aspect when aiming to generate data for a larger number of samples addressing the remaining technical issues such as: antibody quality, array production, sample labelling, and selected assay conditions and biostatistics subjects.

Finally, to address the database issue, Xu et al. [93] constructed the protein microarray database (PMD), which is specifically designed for archiving and analyzing protein microarray data including antibody microarray data. In PMD, users can easily browse and search the entire database by experimental name, protein microarray type, and sample information. Additionally, PMD integrates several data analysis tools and provides an automated data analysis pipeline for users, who can obtain a comprehensive analysis report for their protein microarray data. Making antibody array data available to the community will be one of the important aspects to assess data quality and eventually improve the acceptance of microarray assay data.

## Discussion

### Antibody and affinity reagent libraries

Beyond antibodies, other types of affinity reagents may unlock the bright future of microarrays [78]. Fridy et al. have showed a robust, fast pipeline to produce nanobodies. Inspired by their previous studies of neutralizing HIV antibodies in human, they have developed a strategy for rapid discovery of nanobodies through computation. The nanobody discovery is based on MS identification of affinity-purified heavy-chain antibodies isolated from an individual llama, using a DNA sequence database generated from the same animal. Notably, the authors have provided an advanced technology to improve nanobody’s affinity by conforming dimerized nanobodies to specific antigens. Utilizing this method, phage display could be used to screen for interesting proteins and develop novel nanobody microarrays [94].

Aptamers are single-stranded oligonucleotides, utilized as affinity reagents. Depending on their sequence, the temperature, pH and the presence of certain ions, the aptamers fold into defined three-dimensional (3D) structures [95]. Properly folded aptamers are able to bind other molecules with high affinity and specificity [96]. High-throughput DNA engineering methods can be applied to generate high-diversity libraries to screen for target proteins, which can be utilized in microarray applications. The use of aptamers selected for a slow off-rate has indeed lead to building high-throughput assays for plasma profiling [97], nowadays expanding into the analysis of the plasma proteome in thousands of samples [98]. Recent studies point at an extended use of this platform for studying thousands of proteins and samples. Other affinity reagents, such as designed ankyrin repeat proteins (DARPins) [99] and single-chain variable fragment (scFV) [100, 101] also hold the promise to further boost the advancement and application of affinity reagent microarrays [102].

### Combine with other technologies

#### Flow cytometry

There are further developments and novel applications of antibody microarrays. Sharivkin et al. [103] showed a novel method of combining antibody microarrays with flow cytometry to isolate specific cell types from differentiating stem cell populations. An array of antibodies against cell-surface antigens was printed on a hydrogel coated glass slide. Live cells isolated by different markers using flow cytometry. And then captured on specific antibody spots by interaction between their surface antigens and the printed antibodies. This allowed identifying additional markers that further refined different subpopulation from flow sorting [104].

#### CRISPR/Cas9

The bacterial clustered regularly interspaced short palindromic repeats (CRISPR)-Cas9 system for genome editing has greatly expanded the toolbox for mammalian genetics, enabling the rapid generation of isogenic cell lines and mice with modified alleles. Wang et al. [105] generated a knockout pool for two human cell lines, and this loss-of-function genetic screening approach was suitable for both positive and negative selection. With 1000’s of genes to manipulate in one experiment, the antibody microarray has a great opportunity to be combined with this pool and the CRISPR/Cas9 technology holds promise to create a whole human proteome knock out library, which had the potential to revolutionise how we study proteins and their interactions in health and disease.

#### Advance materials

Chinnasamy et al. presented a novel lateral flow microarray-based device using a novel dually labelled gold nanoparticle-strategy for rapid and sensitive detection of clinical serum samples. Each gold nanoparticle was conjugated to an optimized ratio of HRP and anti-IgE, allowing a significant improvement of assay sensitivity as compared to commercially available detection reagents. Also, due to the rapid and simple procedure, inexpensive materials and read-out by means of a consumer flatbed scanner, the presented assay may provide a sensitive and low-cost platform for multiple fast testing which could facilitate antibody microarray in translation medicine area [106].

Antibody microarrays have been established alongside the advancement of materials. Hu et al. [107] developed a hierarchically nanostructured organic–inorganic hybrid substrate, comprising of randomly oriented ZnO nanorods on glass slides with coaxially tethered dense polymer brush, which highly improved the limit of detection (LOD) to 100 fg/mL. Huang et al. [108] have invented a glycol-gold nanoparticle-based antibody microarray, with potential to amplify a signal that could be detected by the naked eye.

## A perspective on the future applications of antibody microarrays

As outlined above, applications of antibody arrays can be found across various areas of biology and diseases. Some of the concepts have entered clinical testing, while a wide range of content and applications are still being developed or improved. The future of antibody arrays will, among many other factors, depend on three main aspects as eluted to below: (1) validation, (2) continued technical advances, and (3) translation and dissemination.

1. With currently ongoing discussions about the validation of antibodies used for the generation of affinity proteomics assays data, a key element for a continuous use of antibody microarrays will relate to how experimental results from these multiplexed systems can be validated and confirmed by targeted assays. Validation refers to confirming and certifying specificity and reproducibility of the assay across batches of experiments, as well as to describe possible sources of interference and variance. With more and more affinity reagents on the market—currently almost 3,000,000 antibody products can be purchased covering 94% of all human genes (19,155 genes) according to Anitbodypedia (http://www.antibodypedia.org)—not all of these will be functional for antibody microarray assays. Hence, careful upfront qualification and selection of binding molecules is required. This process will require additional, orthogonal and multiplexed assays and read-out systems that resemble the conditions of the array-based assays. An important readout with be the correlation with data from mass spectrometry, as this technology is capable of delivering absolute identifications of target molecules.
2. Analytical sensitivity and degrees of multiplexing are important areas of improvement but these are already common (popular) features that have been addressed ever since. Advances made through interactions with alternative assay concepts and read-out systems are often driven by other areas of life science and engineering. These have and will continue to advance array technologies and its derivate systems. This will also lead to new content being integrated into the current and coming platforms.
3. Lastly, disseminating the method’s utility and data will be key for a continued awareness about the technology. Making antibody array data and assays more available will subsequently enable its use beyond those labs developing technologies to meet and even larger group of users. The success of antibody arrays will then be dependent on how robust and translatable the generated information is. Only if the data provides new leads to advance research projects or supports clinical decision making for an improved precision, then we will see a continued use of antibody arrays.

## Conclusion

In summary, various forms of antibody microarrays have gradually evolved as analytical tools for proteomics research. These microarrays have facilitated advances in basic biology and discoveries towards potential tools of clinical use. With the progressive development, standardization of the experimental workflow and data interpretation, increasing numbers of binding reagents as well as innovative concepts are arising and will provide further advances in the foreseeable future. Antibody microarrays and concepts based upon, hold the promises to contribute to the advancement of research and even diagnostic applications, but there is a strong need to apply the appropriate validation schemes and follow-up studies to confirm the presented indications for a possible clinical decision making.

## Additional file

**Additional file 1.** Detailed information about the home-made and commercially available antibody microarrays.

## Abbreviations

scFvs: single-chain variable fragments
Fab: fragment antigen-binding
CD: cluster of differentiation
ELISA: enzyme-linked immunosorbent assays
DNA: deoxyribonucleic acid
MS: mass spectrometry
CB: calbindin-D28 k
AD: Alzheimer’s disease
mtEF4: mitochondrial elongation factor 4
mTOR: mammalian target of rapamycin-
PDZ: ser-asp-val amino acid
PS-SPCL: positional scanning-synthetic peptide combinatorial libraries
bFGF: basic fibroblast growth factor
PCR: polymerase chain reaction
TNFR1: tumor necrosis factor receptor 1
DD: death domain
PARP: poly ADP-ribose polymerase
MFB: metformin-butyrate
SLE: systemic lupus erythematosus
SSc: systemic sclerosis
ANA: a positive antinuclear antibody
SLEDAI-2k: systemic lupus erythematosus disease activity index 2000
CRP: C-reactive protein
cagA: cytotoxin-associated gene A
HCV: hepatitis C virus
SinFabs: single framework recombinant antibody
IL-9: interleukin-9
IL-11: interleukin-11
MCP-1: monocyte chemoattractant protein 1
MCP-4: monocyte chemotactic protein-4
CA3: carbonic anhydrase III
CK: creatine kinase
AIP: autoimmune pancreatitis
TNF-α: tumor necrosis factor α
AUC: area under the curve
PDA: pancreatic ductal adenocarcinoma
CI: confidence interval
TGF-β: transforming growth factor beta
IFNG: interferon gamma
THBS1: thrombospondin 1
MAPK3: mitogen-activated protein kinase 3
ERK1: extracellular signal-regulated kinase 1
GC: gastric cancer
ICAM-1: intercellular adhesion molecule 1
INPROGAS: inflammatory protein-driven gastric cancer signature
CNDP1: carnosine dipeptidase 1
IGFBP2: insulin like growth factor binding protein 2
IGF1: insulin like growth factor 1
CSF: cerebrospinal fluid
POLG: DNA polymerase gamma
MGMT: O-6-methylguanine-DNA methyltransferase
ApoD: apolipoprotein D
GAP43: growth associated protein 43
NRGN: neurogranin
IRF8: interferon regulatory factor 8
METTL14: methyltransferase like 14
ALS: amyotrophic lateral sclerosis
NEFM: neurofilament medium polypeptide
RGS18: regulator of G-protein signalling 18
SLC25A20: solute carrier family 25
CV: coefficients of variation
ACM: antibody co-localization microarray
DARPins: designed ankyrin repeat proteins
MIAME: minimum information about a microarray experiment
GEO: gene expressiono
SFT: segment and fit thresholding
PMT: photomultiplier tube gain
PMD: protein microarray database
CRISPR -Cas9: clustered regularly interspaced short palindromic repeats
HRP: horseradish peroxidase
IgE: immunoglobulin E
LOD: limit of detection
IHC: immunohistochemistry
FC: flow cytometry
CyTOF: mass cytometr
